# Reduced inclination of cervical spine in a novel notebook screen system - implications for rehabilitation

**DOI:** 10.1186/1745-6673-6-30

**Published:** 2011-11-25

**Authors:** David Quarcoo, Cristian Scutaru, Ulrich Henkel, Michael F Spallek, Stefanie Uibel, Karin Vitzthum, Stefanie Mache, Bianca Kusma, David A Groneberg

**Affiliations:** 1 Institute of Occupational Medicine, Charité - Universitätsmedizin Berlin, Free University and Humboldt University, D-14195 Berlin, Germany; 2 Department of Respiratory Medicine, Hannover Medical School, Hannover, Germany

## Abstract

**Background:**

Professional working at computer notebooks is associated with high requirements on the body posture in the seated position. By the high continuous static muscle stress resulting from this position at notebooks, professionals frequently working at notebooks for long hours are exposed to an increased risk of musculoskeletal complaints. Especially in subjects with back pain, new notebooks should be evaluated with a focus on rehabilitative issues.

**Methods:**

In a field study a new notebook design with adjustable screen was analyzed and compared to standard notebook position.

**Results:**

There are highly significant differences in the visual axis of individuals who are seated in the novel notebook position in comparison to the standard position. Also, differences are present between further alternative notebook positions. Testing of gender and glasses did not reveal influences.

**Conclusion:**

This study demonstrates that notebooks with adjustable screen may be used to improve the posture. Future studies may focus on patients with musculoskeletal diseases.

## Introduction

**Figure 1:**
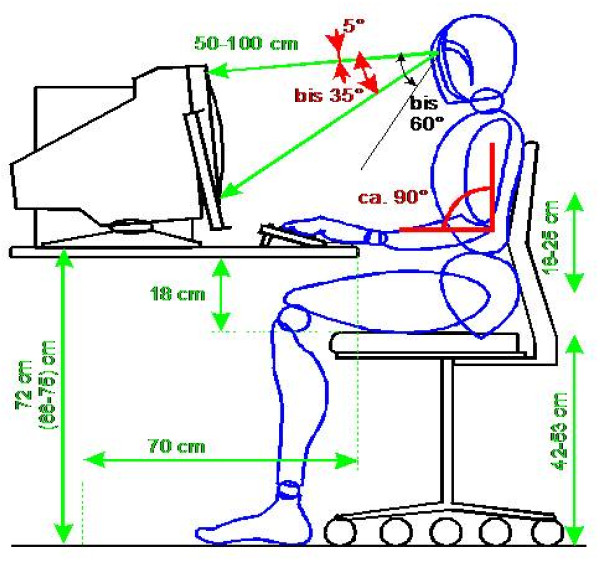
**Postural requirements according to the national regulation for workstation (BildscharbV)**.

Over the past centuries a profound change in the work reality of most citizens has happened worldwide. In Europe at the end of the 19^th ^century great parts of the workforce was employed in the agriculture and producing sector [1]. Today these sectors cease importance in regard to the people employed while the service and information industries have gained importance [1]. Here the typical work environment is the workstation. Although the fading away of the heavy and dirty work lead to an exoneration of health risks new work related health challenges have appeared. Above all the psychological and ergonomic burden of work gets into the focus of interest [2]. The physical strain of the office work relates to the musculoskeletal system which centers on the shoulder/arm and cervical and lumbar region [3]. Local strain in the musculoskeletal system can be relayed to distant sites, resulting in complains in further regions. Diseases of the musculoskeletal system are a frequent cause of work related morbidity. In a recent survey of the Federal Institute for Occupational Safety and Health 46,2% of the total workforce experienced shoulder neck pain, of which 61% lead to medical consultation [4]. The study identified declination of the cervical spine a risk factor next to forced posture and hard labor. In this line detailed specifications have been worked out to minimize work related adverse effects at these workplaces. In Germany extended research has lead to a national regulation for workstation (BildscharbV) that defines the workstation delineating sizes of chair and table to guarantee optimal body and visual axis [5]. This approach introduced a (average) body size independent evaluation of the workplace (Figure 1).

The increasing mobility together with growing work intensification results in the desire to use traveling time to continue computer based work. Therefore the amount of mobile computer units such as notebooks etc. has increased over time [6]. Owing to the compact design these units do not comply with above mentioned guide lines. Docking station and extern keypads might alleviate some concerns but the problem of an unfavorable visual axis remains.

Owing to the difficult ergonomic situation notebooks might easily become a hindrance for productivity and a potential problem for the well being and health [7].

Recently this problem was addressed by the novel design of the notebook lid that allows the extension of the screen in a vertical plane. The concept envisions that in operation modus the screen is lifted various steps upwards and locked in place to allow a more extended position of the cervical spine.

The aim of the current study was to analyze characteristics of a note book with a variable extended screen system. We hypothesize that the new system leads to a lower degree of inclination and may therefore serve for rehabilitative issues

## Materials and methods

### Study population

Healthy probands were recruited by public notice. Information on health was collected by questionnaire. Thirty test persons with written consent, fulfilling the criteria were selected into the study. The subjects' anthropometric characteristics are presented in detail in Table 1. The primary inclusion criterion for the study was no severe disease or trauma of the musculoskeletal system. Other inclusion criteria were age between 20 and 60 years.

**Table 1:** Anthropomorphic data of test persons

Gender	Average age	Average height	Average weight	Corrective lenses
Female	32.50 y.	169.25 cm	62.00 kg	12/30

Male	31.22 y.	184.0 cm6	84.28 kg	18/30

### Determination of cervical flexion

**Figure 2:**
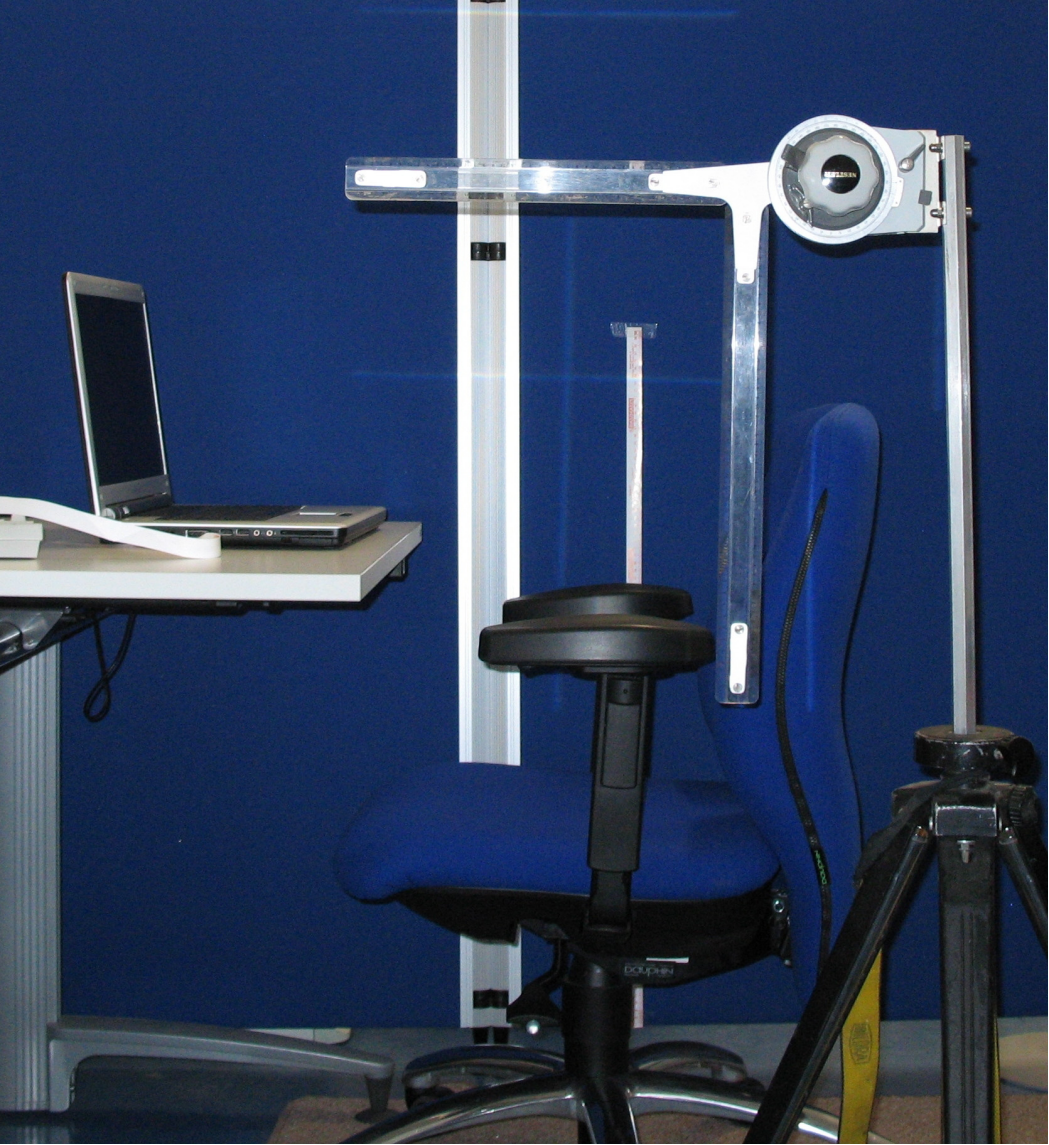
**Study set-up**. The workstation with adjustable ergonomic chair and desk as well as the adaptable protractor are displayed.

**Figure 3:**
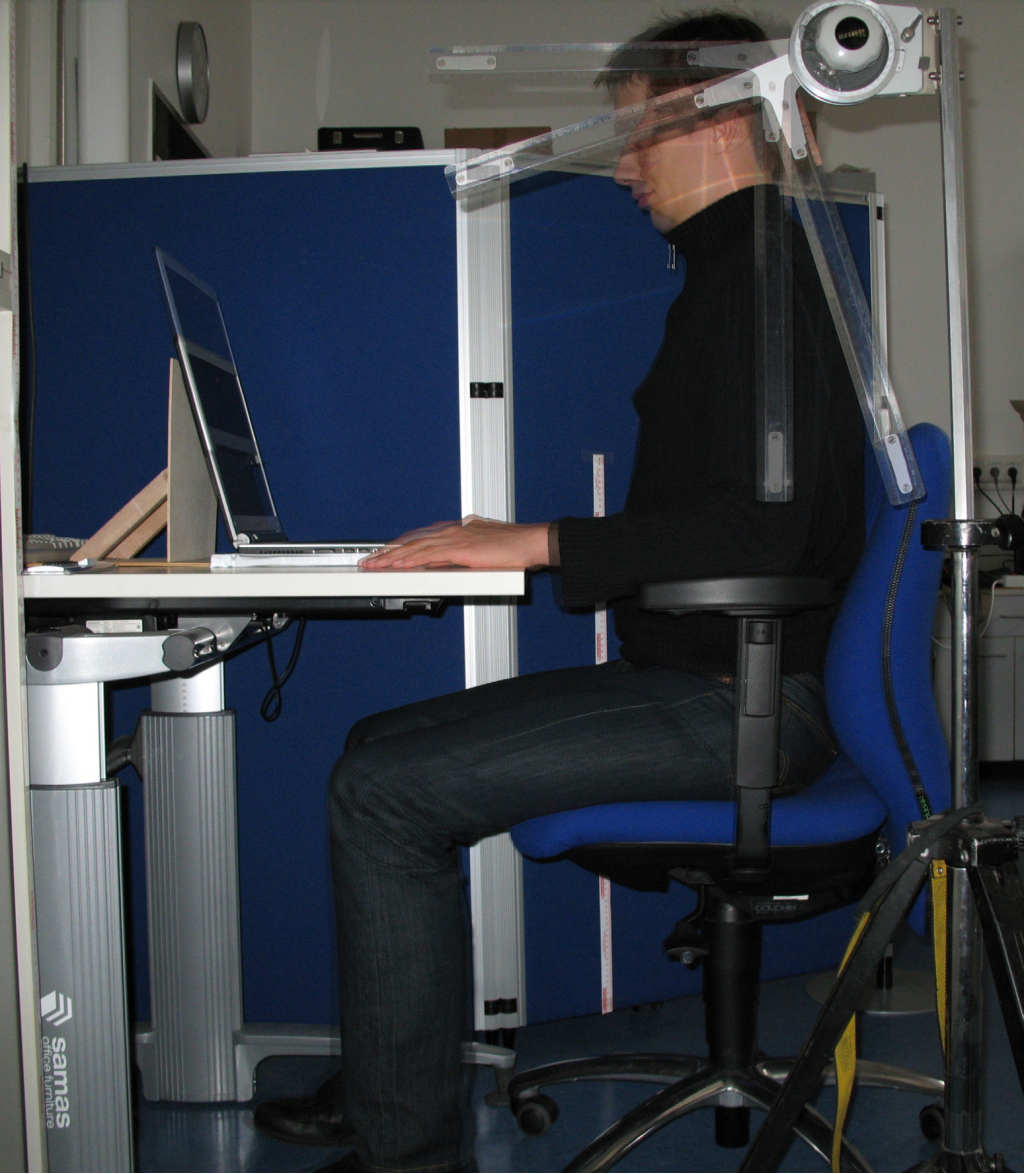
**Optical measurement of the visual axis in photo digital overlay technique**.

Subjects were positioned on a height adjustable office chair and desk in compliance with the national guideline for workstations. The inclination of the cervical spine was measured in different positions in reference to the visual axis using an adaptable protractor mounted on a stand (Figure 2). When determining the inclination, the test operator verified the eye position to be in an intra-study comparable middle position. After a position with straight visual axis, position of the computer screen was adjusted according to a test routine described in Table 2 and inclination of the cervical spine was measured. A notebook with 15 inch screen was used. Digital photo overlay techniques were applied to compare the positions (Figure 3).

**Table 2:** Different experimental positions

Position 0	Straight visual axis, without gazing to the computer screen.
Position 1	Maximal extension of computer screen (38 cm upper edge of screen)

Position 2	Second extension of computer screen (33 cm upper edge of screen)

Position 3	Second extension of computer screen (31 cm upper edge of screen)

Position 4	Common notebook screen position (27 cm upper edge of screen)

### Ethics

The local ethics committee approved the study and the participants gave their informed written consent prior to inclusion in the study.

### Statistics

Results are expressed as means with standard deviations (SD). Due to the small sample size non-parametric methods were used, because they are more robust. Φ was used instead of the chi-square distribution if the frequencies were too low (more than 20% of the cells had an expected count less than 5) to avoid type II errors. A p-value of less than .05 was considered significant. Analyses were performed using SPSS version 17.0.

## Results

### Optical measurements

The inclination was determined using optical measurements. To verify the results a photo digital overlay technique was used that demonstrated substantial differences in the various positions (Figure 3).

### Cervical inclination in different positions

**Figure 4:**
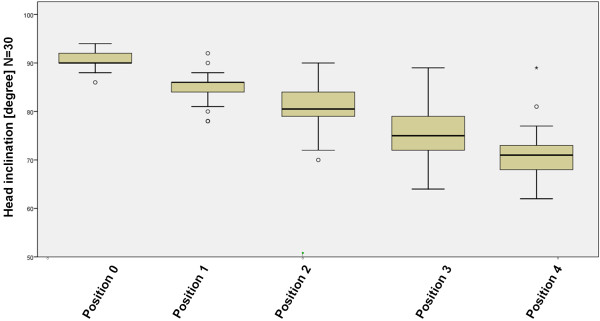
**Cervical inclination in different positions**. The cervical inclination of all individuals (n = 30) is depicted in the different screen positions. Significances are displayed in Table. 3.

All test persons were measured in all 5 positions (Figure 4). The cervical inclination was 90,3° in the position 0, 85,03° in position 1, 80,4° in position 2, 75,5° in position 3 und 71,53° in Position 4 (Figure 4). The statistical analysis resulted in significant differences between the different positions 0-4 (Table 3).

**Table 3:** Differences between the experimental positions

	Position 0	Position 1	Position 2	Position 3	Position 4
Position 0	X	X	X	X	X

Position 1	φ = 1.7*	X	X	X	X

Position 2	φ = 1,6	φ = 2.2	X	X	X

Position 3	φ = 1,9	φ = 2.5	φ = 2.8**	X	X

Position 4	φ = 1.8	φ = 2.6**	φ = 2.7**	φ = 2.9*	X

*p < 0.05, **p < 0.01

### Gender influence on cervical inclination

**Figure 5:**
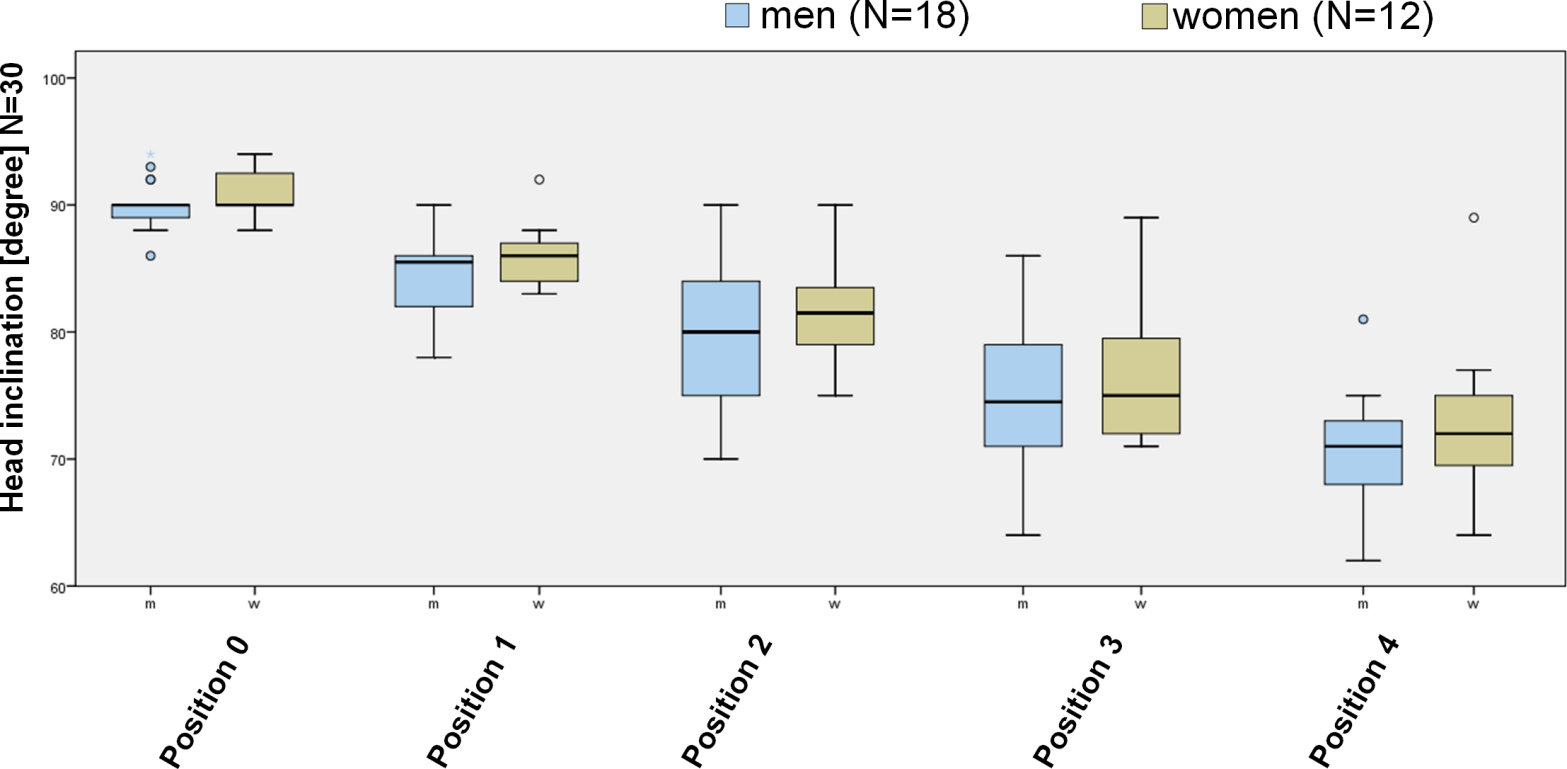
**Influence of gender**. The cervical inclination of female and male individuals is depicted in the different screen positions.

The influence of gender was determined for the 5 positions (Figure 4). We found that men (18) had a cervical inclination of 90,06° in Position 0, 84,44° in position 1, 79,61° in position 2, 74,78° in position 3 und 70,61° in position 4 (Figure 5). The inclination in female test persons (12) was 90,67° in position 0, 85,92° in position 1, 81,58° in position 2, 76,58° in position 3 und 72,92° in position 4. As for the total test population a difference was found within a gender group between positions. The gender group did not differ significantly in each of the positions.

### Influence of corrective lenses on cervical inclination

**Figure 6:**
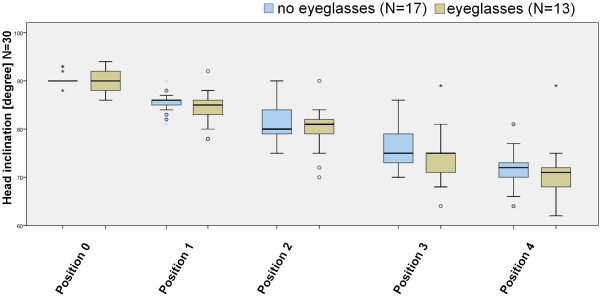
**Influence of correction lenses**. The cervical inclination individuals with/without correction lenses is depicted in the different screen positions.

The influence of the usage of corrective lenses on the cervical inclination was investigated for all 5 positions. The average inclination of subjects without corrective lenses was 90,35° in position 0, 85,53° in position 1, 80,88° in position 2, 76,29° in position 3 und 71,82° in position 4 (Figure 6). The data for probands with corrective glasses (13) demonstrated a cervical inclination of 90,23° in position 0, 84,38° in position 1, 79,77° in position 2, 74,46° in position 3 und 71,15° in position 4. There was no significant difference between the two groups in each of the positions.

## Discussion

The increasing mobile use of notebooks poses a problematic ergonomic situation. To circumvent some of the negative effects on the musculoskeletal system that occurs with the unfavorable body position a novel screen system was designed. This height adjustable display was evaluated in the current study. The cervical inclination that corresponded to the five position of the computer display differed significant in all subjects resulting in a reduced flexion in the maximum moved out position.

The vertical strain on the spine is reflected by the force that acts on the intravertebral discs that lead to changes in the intradiscal pressure (PID). It has been suggested that an increased PID may worsen the alimentary status of the intravertebral disc that might contribute to a faster advancing of degenerative processes [8-10]. Studying the lumbar spinal region Nachemson and coworker demonstrated that different body postures influence the intradiscal pressure [11,12]. The results were confirmed by data from discography and chemonucleolysis [10]. There are important differences between the sections of the spine. In cervical discs, the nucleus is less able to equalize stress over large distances, and the posterior annulus does not sustain high compressive stresses [13]. Although most research focused on the lumbar spine, recent data has found a postural dependence also for the cervical spine [8,9]. PID is lowest in the middle position between flexion and extension [9]. This relation has found expression in national guidelines where an only marginal flexion of the neck with the least stress is favored for the working environment.

Next we evaluated factors that might influence the extent of inclination. In this context Nightingale and coworker have found gender specific anatomic differences of the cervical spine. The male upper cervical spine is significantly stiffer and stronger [14]. In our study population there was no difference between the neck inclination of the male and female subjects.

Also for another possible influencing factor - the wearing of correction lenses - no distinction was found, supporting the relevance of the data.

In summary the moved out position of a new height adjustable notebook display reduces significantly the cervical inclination. From data that the vertical strain on the cervical spine depends on the degree of inclination, it may be assumed that in this position the strain is reduced. The novel screen advances the notebook display ergonomically next to desktops. It may be especially beneficial in rehabilitation.

Future studies will evaluate the novel displays' ability to reverse already set in damages of the musculoskeletal system. Furthermore the influence on the upper body and the position of the keyboard will be future research topics.

## Conflict of interests

This study was funded by Dreamcom Deutschland GmbH. The views in this article are the personal views of the authors and do not necessarily represent the views of the professional organizations or institutions within which we are members.

## Authors' contributions

DAG, UH and DQ drafted the manuscript. DAG, CS, UH and DQ conceived the study and the study design, performed the analysis and interpretation of the data. MFS, SU, KV, SM, BK: Participation in the analysis of data, revision of the manuscript. All authors read and approved the final manuscript.
